# Social Company by a Receptive Mating Partner Facilitates Fear Extinction

**DOI:** 10.3389/fnins.2020.00062

**Published:** 2020-02-07

**Authors:** Feng Gao, Jie Huang, Yan-Fei Guan, Guo-Bin Huang, Wen-Jing Li, Xi-Yi He, Zi-Cong Qiu, Yun-Long Zhang, Shen-Ting Zhao, Jianhua Li, Aiguo Xuan, Xiang-Dong Sun

**Affiliations:** ^1^School of Basic Medical Sciences, Institute of Neuroscience and the Second Affiliated Hospital of Guangzhou Medical University, Key Laboratory of Neurogenetics and Channelopathies of Guangdong Province and the Ministry of Education of China, Guangzhou, China; ^2^KingMed School of Laboratory Medicine, Guangzhou Medical University, Guangzhou, China; ^3^The First School of Clinical Medicine, Guangzhou Medical University, Guangzhou, China; ^4^Key Laboratory of Protein Modification and Degradation, School of Basic Medical Sciences, Affiliated Cancer Hospital & Institute of Guangzhou Medical University, Guangzhou, China; ^5^Guangdong Province Key Laboratory of Psychiatric Disorders, Guangzhou, China

**Keywords:** post-traumatic stress disorder, exposure therapy, fear extinction, social company, BLA

## Abstract

Fear extinction remains an unresolved challenge for behavioral exposure therapy in patients with post-traumatic stress disorder (PTSD). Previous reports have suggested that social support from either familiar or unfamiliar same-sex partners is beneficial to attenuating fear responses during fear extinction and renewal. Despite that, few studies have examined the effects of social support in advance on fear extinction and/or retrieval. It is also not clear whether social company by a receptive mating partner in advance facilitates fear extinction. In the present study, we address these questions by introducing a co-housing method, where fear-conditioned male mice are co-housed with or without a receptive mating partner prior to fear extinction. We found that while co-housing with an ovariectomized female mouse showed little effect on fear extinction or retrieval, social company by a receptive mating partner in advance dramatically facilitates fear extinction. In addition, the number of cFos-positive neurons in the basolateral amygdala (BLA) were also found to be reduced in male mice accompanied with receptive mating partner in response to fear extinction and retrieval, indicating diminished neuronal activation. Electrophysiological studies further showed that the excitability of excitatory neurons in BLA was decreased, which is probably due to the attenuated basal level of excitatory synaptic transmission. Together, our observations demonstrate an effect of social company by a receptive mating partner can facilitate fear extinction and afford a possible cellular mechanism.

## Introduction

Post-traumatic stress disorder (PTSD) is a serious mental disorder with lifetime prevalence that ranges from 1.3 to 12.2% (Shalev et al., 2017). PTSD usually happens to individuals who have been suffering from a traumatic environmental event, and it is characterized by intrusive symptoms, avoidance behaviors, negative alterations in mood and cognition, as well as hypervigilance (Pitman et al., 2012). Clinically, “exposure” therapy is the common treatment method utilized to treat PTSD patients. It involves repeatedly exposing patients to trauma-related stimuli in a safe way in order to help them overcome their traumatic experience (American Psychiatric Association [APA], 2013). Nevertheless, some patients failed to show significant improvement from the exposure therapy, and the underlying reasons are not clear (Sillivan et al., 2017; Mayo et al., 2018).

In the last decades, despite efforts from multiple studies, which were designed to elucidate the mental and biological foundations of PTSD, the underlying pathological mechanisms remain obscure (Tovote et al., 2015). In rodents, the Pavlovian fear extinction model is often used to simulate exposure therapy for PTSD. In this fear extinction paradigm, animals are first trained to achieve fear memory by pairing a neutral auditory stimulus (conditioned stimulus, CS) with an aversive foot shock (unconditioned stimulus, US). During a fear extinction session, animals were subjected to a novel context with repeated exposure to CS alone (Milad and Quirk, 2002).

Results from previous studies have supported the notion that the “social buffering” effect exists not only in humans but also in non-human primates as well as rodents (Tol et al., 2011; Lieberwirth and Wang, 2016). This phenomenon usually results in better recovery from aversive experiences when they are accompanied by conspecific partners. Multiple findings have also consistently demonstrated that social support from either familiar or unfamiliar same-sex partners can alleviate symptoms of PTSD, such as through reductions in the level of anxiety and fear response (Gros et al., 2016; Soykoek et al., 2017). For example, the presence of an unfamiliar same-sex rat during extinction dramatically reduced the fear response and hypothalamic-pituitary-adrenal (HPA) axis activation in the fear-conditioned rats (Mikami et al., 2016). Social company provided by an unfamiliar same-sex partner during either fear extinction or the test stage was also found to be effective in the prevention of fear renewal outside of the extinction context (Yuan et al., 2018). Since the conspecific partner only appeared during extinction or renewal in both studies, it would therefore be interesting to know whether social support in advance can also facilitate fear extinction and/or retrieval.

Social support is available from friends and family members in one’s social environment, especially intimate partners since they may impose significant influence over the symptoms and treatment outcome in PTSD patients (Laffaye et al., 2008; Pietrzak et al., 2010). While the affinity and attachment to accompanying conspecifics are regarded as the main basis for social buffering (Kiyokawa et al., 2014), sexually matured males are usually prone to targeting their attachment and affiliation to a mating partner (Kiyokawa, 2017). However, to date, very few studies have examined the influence of the social support from a mating partner prior to fear extinction on fear memory.

In the present study, we addressed these issues by introducing a co-housing method. We first subjected the adult male mice to intense fear conditioning. After acquiring a strong fear memory, male mice were individually housed either alone (FC alone group), with an ovariectomized female mouse (FC + OVC group), or with a receptive female mouse (FC + Receptive group). Twenty-four hours later, all male mice were individually subjected to fear extinction training as well as a retrieval test on the next day. To examine the underlying mechanisms, we characterized neuronal activation in the basolateral amygdala (BLA) by using cFos staining and recorded neuronal excitability and excitatory synaptic transmission by using a patch clamp technique.

## Materials and Methods

### Reagents and Antibodies

Chemicals were purchased from Sigma-Aldrich unless otherwise indicated. Mouse monoclonal anti-cFos and rabbit polyclonal anti-PV primary antibodies were obtained from Santa Cruz (sc-271243; 1:100 for staining) and Swant (PV27; 1:5000 for staining), respectively.

### Animals

The experiments followed the Guidelines for Animal Care and Use of China, and the experimental protocols were approved by the Animal Ethics Committee of Guangzhou Medical University (Ou et al., 2018). Significant efforts were made to reduce the number of animals and minimize their suffering during the experiments.

Adult C57BL/6J mice aged 9–10 weeks were purchased from Guangdong Medical Laboratory Animal Center. Male mice were individually housed, and female mice were housed in groups of four for at least 1 week prior to experiments. Mice were maintained in a temperature- and humidity-controlled holding facility with 12-h light/dark cycles (lights on from 08:00 to 20:00). Food and water were available *ad libitum*.

### Ovariectomy

Adult female mice were anesthetized with 1% pentobarbital sodium. The ovaries, located in a fat pad beneath the muscles, were exposed through bilateral flank incisions. With the help of forceps, the oviducts were ligated, and ovaries were exteriorized. The muscle of the posterior abdominal wall and the exterior skin incision were then sutured. After the surgery, mice were injected with ketoprofen (5 mg/kg) subcutaneously to deliver analgesia. Mice were returned to their home cages, monitored daily, and provided with a recovery period of at least 7 days before experiments.

### Preparation of Receptive Female Mice

Receptive female mice were prepared as described previously (McHenry et al., 2017). Briefly, ovariectomized female mice (OVC) were administered subcutaneously with estradiol at 0.02 mg and progesterone at 0.5 mg in 0.05 ml of sesame oil 48 and 4 h prior to co-housing. Receptive female mice were then transported into cages with fear-conditioned male mice.

### Fear Conditioning and Extinction

All subjects were handled for 3 days before behavioral experiments. Prior to fear conditioning, male mice were individually housed. Paradigms that measure tone-cued fear memory formation and extinction were chosen according to a recent report in which fear memory was hardly degraded under such paradigms (Adhikari et al., 2015). Briefly, tone-cued fear conditioning was conducted in the sound-attenuating chamber with a metal grid floor connected to a shock generator (Med Associate Inc.). On day 1, after a 2-min acclimation period (Baseline) in Context A (metal grid floor, interior white light, fans, and mild alcohol scent), male mice were exposed to six conditioned tone stimuli (CS: 20-s duration, 2.9 kHz, 85 dB) that were each co-terminated with a foot shock (US: 0.7 mA, 2-s duration) at pseudo-random inter-trial intervals (∼2 min on average). Freezing level was calculated as the percentage of time spent in a freezing posture in the presence of the tone. Chambers were cleaned with 70% ethanol at the end of each trial.

After fear conditioning, each mouse was immediately returned to its home cage. They were divided into three groups based on their freezing level in the training phase, ensuring a similar freezing level prior to subsequent manipulations: Group 1: male mouse without a partner (FC alone); Group 2: male mouse accompanied with an ovariectomized female mouse (FC + OVC); and Group 3: male mouse accompanied with a receptive female mouse (FC + Receptive). Twenty-four hours later, male mice were subjected to fear extinction procedure.

For fear extinction, each male mouse was transported to a chamber with an altered environment (Context B: flat floor, rounded wall, and mild acetic acid scent) on day 2. The freezing level was measured for 2 min without CS (Baseline, BL) and in the presence of subsequent 10 CS with an interval time of 90 s between each CS. The formula for deriving the% cued freezing used was (freezing time in the presence of a tone/total time of a tone) × 100%. For the retrieval test, male mice were placed again in the same chamber as in day 2 (Context B) on day 3 for 2 min prior to CS. Then, a single CS (tone, 20 s) was presented. Freezing time was recorded in the absence and the presence of CS. Chambers were cleaned with 1% acetic acid solution at the end of each trial.

### Immunofluorescence

As described previously (Gong et al., 2019; Kong et al., 2019), male mice were anesthetized with 1% pentobarbital sodium and perfused intracardially with saline, followed by 4% paraformaldehyde in 0.1M phosphate buffer in 1 h after fear retrieval test. The brain was removed and immersed overnight in 4% paraformaldehyde in 0.1M phosphate buffer and then placed in 30% sucrose/phosphate buffer for cryoprotection. Six successive 30-μm sections containing the BLA were sectioned with a freezing microtome (CM1950, Leica). After being permeabilized with 1% Triton-100 and 5% bovine serum albumin (BSA) in 0.01M PBS, sections were incubated with primary antibodies overnight at 4°C. After washing the sections three times with PBS (each for 10 min), they were incubated with Alexa Fluor-conjugated secondary antibodies (1:1000, Jackson laboratory, donkey anti-mouse Alexa Fluor 488 for cFos staining; donkey anti-rabbit Alexa Fluor 594 for PV staining) for 1 h. Following three times of washing with PBS (each for 10 min), slices were cover-slipped with fluoroshield mounting medium with 4′, 6-diamidino-2-phenylindole (ab104139; Abcam). For immunohistochemical analyses, images of the BLA sections were captured using a LSM800 confocal microscopy (ZEISS). An experimenter who was blind to the experimental groups counted the number of cFos-positive and PV-positive cells within a 0.5-mm square by means of ImageJ software.

### Slice Preparation

Slices containing BLA were prepared as described previously (Sun et al., 2018; Huang et al., 2019). Briefly, mice were anesthetized with isoflurane and subsequently decapitated. Brains were quickly removed and placed in ice-cold oxygenated modified artificial cerebrospinal fluid (ACSF) containing 120 mM choline chloride, 2.5 mM KCl, 0.5 mM CaCl_2_-2H_2_O, 2.5 mM MgSO_4_, 1.25 mM NaH_2_PO_4_, 7 mM MgCl_2_, 25 mM NaHCO_3_, and 10 mM glucose. Brain slices (300 μm) were cut in ice-cold modified ACSF with a VT-1000S vibratome (Leica, Germany) and subsequently transferred to a storage chamber containing regular ACSF (126 mM NaCl, 3 mM KCl, 1.25 mM NaH_2_PO_4_, 2.0 mM CaCl_2_, 1.0 mM MgSO_4_, 26 mM NaHCO_3_, and 10 mM glucose) for a 30-min recovery period at 32°C and followed at room temperature (25 ± 1°C) for an additional 1 h before recording. All solutions were saturated with 95% O_2_/5% CO_2_ (vol/vol).

### Electrophysiological Recordings

Slices were placed in the recording chamber that was perfused (2 ml/min) with ACSF at room temperature (25 ± 1°C). Whole-cell patch-clamp recordings of excitatory neurons in BLA were visualized with infrared optics using an upright microscope equipped with an infrared-sensitive CCD camera (DAGE-MTI, IR-1000E) and a 40× water immersion lens (Olympus). The pipettes were pulled by a micropipette puller (P-97, Sutter instrument) with a resistance of 3–5 MΩ. Recordings were made with a MultiClamp 700B amplifier and 1440A digitizer (Molecular Device). Glass pipettes (3–5 MΩ) were filled with a solution containing 125 mM potassium gluconate, 5 mM KCl, 10 mM HEPES, 1 MgCl_2_, 4 mM Mg-ATP, 0.3 mM Na-GTP, 10 mM disodium phosphocreatine, and 0.2 mM EGTA, pH 7.4 with KOH, 285–300 mOsm. Excitatory neurons in the BLA region were identified by their pyramidal shape and spike frequency adaptation induced by a prolonged depolarizing current injection. To assess the neuronal excitability, action potentials (APs) were measured by injecting a series of depolarizing pulses (from 0 to 140 pA at a step of 20 pA, 10 s as interval) under a current-clamp mode. Resting membrane potential and membrane input resistance were also calculated in response to a series of hyperpolarizing pulses. Spontaneous excitatory postsynaptic currents (sEPSCs) were recorded from the excitatory neurons at holding potential of −70 mV and in the presence of 20 μM GABAa receptor blocker-bicuculline methiodide (BMI). Data were filtered at 1 kHz and sampled at 10 kHz. Neurons were collected when the series resistance fluctuated within 20% of the initial values (<20 MΩ) and analyzed using pClamp 10.2 software for neuronal excitability and MiniAnalysis for sEPSCs.

### Statistical Analysis

Data are illustrated as mean ± standard error of the mean (SEM), and the number of experimental animals is indicated by “*n.*” All statistical analyses were performed using a GraphPad Prism7. The sample size choice was based on previous studies (Lu et al., 2014; Sun et al., 2016), and it was not predetermined by a statistical method. No randomization method was used. Data distribution was assumed to be normal, but this was not formally tested. A student’s *t*-test was used to compare data from two groups. A one-way ANOVA with repeated measures was used in the fear extinction study that analyzed freezing level in response to tones for each group. A repeated two-way ANOVA was used in the fear extinction studies, while a regular two-way ANOVA was used in the electrophysiological studies that analyzed no less than two parameters. The significance threshold was set as ^∗^*P* < 0.05, ^∗∗^*P* < 0.01, and ^∗∗∗^*P* < 0.001 for all results.

## Results

**FIGURE 1:**
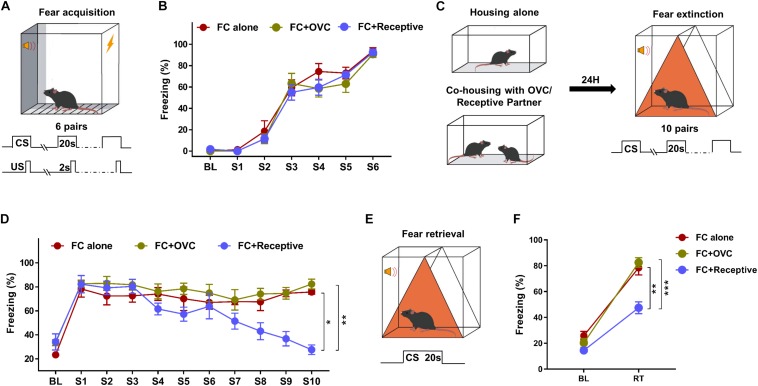
Social company by a receptive mating partner facilitates fear extinction. **(A)** Schematic of the tone-cued fear conditioning procedure. Six pairs of US (Foot shock, 0.7 mA, 2 s)–CS (tone, 20 s) were delivered during fear condition training. **(B)** A robust freezing response was elicited by the CS during fear conditioning. *n* = 8 mice for each group. BL, baseline; S, tone. **(C)** Diagram of co-housing design and schematic of the tone-cued fear extinction training. CSs were delivered 10 times with an interval time of 90 s. **(D)** Gradually reduced freezing level of male mice in the “FC + Receptive” group. *n* = 8 mice for each group. One-way ANOVA: ***P < 0.001. Repeated two-way ANOVA, group effect: “FC + Receptive” vs. “FC alone”, ^∗^*P* = 0.0184; “FC + Receptive” vs. “FC alone,” ^∗∗^*P* = 0.0016. **(E)** Diagram of extinction retrieval test. Freezing time was recorded in the absence and presence of CS. **(F)** Freezing level was decreased in male mice in the “FC + Receptive” group in retrieval test. *n* = 8 for each group. Student’s *t*-test: “FC + Receptive” vs. “FC alone,” **P = 0.001; “FC + Receptive” vs. “FC alone,” ***P < 0.001.

During the intense fear conditioning training with six pairs of CS–US (Figure 1A), adult male mice exhibited a robust freezing response (92.25 ± 1.69%; Figure 1B). To determine whether company by a receptive mating partner in advance facilitates fear extinction, a portion of male fear-conditioned mice were individually accompanied by a receptive female mouse 24 h prior to fear extinction (Figure 1C, FC + Receptive group). Fear conditioned male mice without partners (FC alone group) or co-housed with OVC (FC + OVC group, unable to mate) were used as controls. During fear extinction training, the freezing levels in response to the first tone (S1) were not significantly different among the three groups (Figure 1D, Student’s *t*-test, “FC + Receptive” vs. “FC alone,” *t*(14) = 0.4009, *P* = 0.6946; “FC + Receptive” vs. “FC + OVC,” *t*(14) = 0.0543, *P* = 0.9574; “FC + OVC” vs. “FC alone,” *t*(14) = 0.5926, *P* = 0.5629), which was suggestive of similar fear expression and the limited effects of co-housing on fear consolidation. In accordance with a previous report (Mikami et al., 2016), the fear extinction procedure containing 10 consecutive CS hardly decreased the freezing levels of male mice in “FC alone” and “FC + OVC” groups (Figure 1D; One-way ANOVA, “FC alone”: *F*_(__9_,_63__)_ = 0.5238, *P* = 0.8519; “FC + OVC”: *F*_(__9_,_63__)_ = 0.8912, *P* = 0.5383). In addition, the freezing level was not found to be significantly different between these two groups (repeated two-way ANOVA, group effect, *F*_(__1_,_126__)_ = 1.67, *P* = 0.2172). The interaction between group and trial was also found to be not significant (*F*_(__9_,_126__)_ = 0.2375, *P* = 0.9883). These results suggest that the presence of a non-receptive partner (ovariectomized female) prior to fear extinction exhibits little effect on fear extinction. However, the freezing levels of male mice in the “FC + Receptive” group were gradually reduced during the extinction procedure (Figure 1D; One-way ANOVA, *F*_(__9_,_63__)_ = 11.84, *P* < 0.001). When compared with those in “FC alone” and “FC + OVC” groups, the freezing levels in the “FC + Receptive” group were dramatically reduced (repeated two-way ANOVA, group effect, “FC + Receptive” vs. “FC alone”: *F*_(__1_,_126__)_ = 7.116, *P* = 0.0184; “FC + Receptive” vs. “FC + OVC”: *F*_(__1_,_126__)_ = 15.23, *P* = 0.0016). The interactions between group and trial were also significant (“FC + Receptive” vs. “FC alone”: *F*_(__9_,_126__)_ = 6.1, *P* < 0.0001; “FC + Receptive” vs. “FC + OVC”: *F*_(__9_,_126__)_ = 5.612, *P* < 0.0001). Collectively, these observations indicate facilitated acquisition of fear extinction in the “FC + Receptive” group. During a retrieval test (Figure 1E), mice in both the “FC alone” and “FC + OVC” groups exhibited elevated freezing levels in response to CS, and the freezing levels were comparable between the two groups (Student’s *t*-test, *t*(14) = 0.617, *P* = 0.547). However, the freezing level of male mice in the “FC + Receptive” group was dramatically decreased when compared against “FC alone” and “FC + OVC” groups (Figure 1F; Student’s *t*-test, “FC + Receptive” vs. “FC alone,” *t*(14) = 4.312, *P* = 0.001; “FC + Receptive” vs. “FC + OVC,” *t*(14) = 5.899, *P* < 0.001). Please note that the reasons for the reduced freezing level in the retrieval test are not clear since the differences in acquisition of extinction may underlie subsequent differences in retrieval, and the effects of sexual activity on fear retrieval were not specifically determined in the present study. Nevertheless, these observations demonstrate that company by a receptive mating partner, but not a non-receptive one, in advance facilitates acquisition of fear extinction.

**FIGURE 2:**
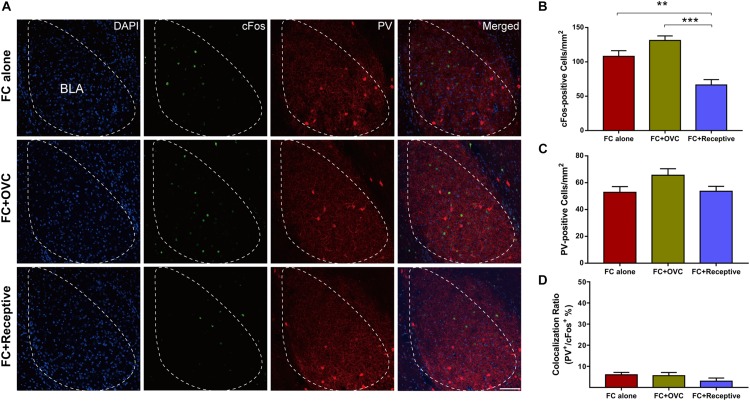
Reduced neuronal activation in the BLA by co-housing with a receptive mating partner. **(A)** Representative images of cFos and PV staining in the BLA. Scale bar,100 μm. **(B)** The number of cFos-positive neurons in the BLA was decreased in the “FC + Receptive” group. *n* = 5 mice for each group. Student’s *t*-test, “FC + Receptive” vs. “FC alone”: **P = 0.0064; “FC + Receptive” vs. “FC + OVC”: ***P < 0.001. **(C)** Similar densities of PV-positive neurons in the BLA among three groups. Student’s *t*-test, *P* > 0.05. **(D)** Little colocalization ratio of PV and cFos-positive neurons in the BLA (Ratio: “FC alone” = 6 ± 1.14%; “FC + OVC” = 5.6 ± 1.50%; “FC + Receptive” = 3 ± 1.48%).

It has been reported that the amygdala is a vital brain area correlated with the social and fear-related behaviors (Bickart et al., 2011). In particular, it was found that BLA actively participated in fear extinction processing, and the aberrant neuronal activation in the BLA was implicated in enhanced fear extinction (Bocchio et al., 2017; Davis et al., 2017). Thus, we hypothesized that neuronal activation in the BLA may be decreased by co-housing with a receptive mating partner. To verify this hypothesis, we characterized the cFos-positive neurons in the BLA region of male mice from each group after the fear retrieval test. We found that the number of cFos-positive neurons in the “FC + Receptive” group was reduced compared to those in the “FC alone” and “FC + OVC” groups (Figures 2A,B; Student’s *t*-test, “FC + Receptive” vs. “FC alone,” *t*(8) = 3.663, *P* = 0.006; “FC + Receptive” vs. “FC + OVC,” *t*(8) = 6.354, *P* < 0.001). This result suggests reduced neuronal activation in the BLA of mice accompanied by a receptive mating partner. We also examined the parvalbumin (PV)-positive interneurons in the BLA region since they are the foremost interneurons in the BLA and modulate excitatory neurons in fear extinction processing (Ehrlich et al., 2009; Trouche et al., 2013). As shown in Figure 2C, the number of PV-positive interneurons are similar among the three groups (Student’s *t*-test, “FC + Receptive” vs. “FC alone,” *t*(8) = 0.1414, *P* = 0.891; “FC + Receptive” vs. “FC + OVC,” *t*(8) = 1.97, *P* = 0.0844; “FC + OVC” vs. “FC alone,” *t*(8) = 1.985, *P* = 0.0825), indicating unaltered inhibitory neuron densities in mice accompanied by a receptive mating partner.

**FIGURE 3:**
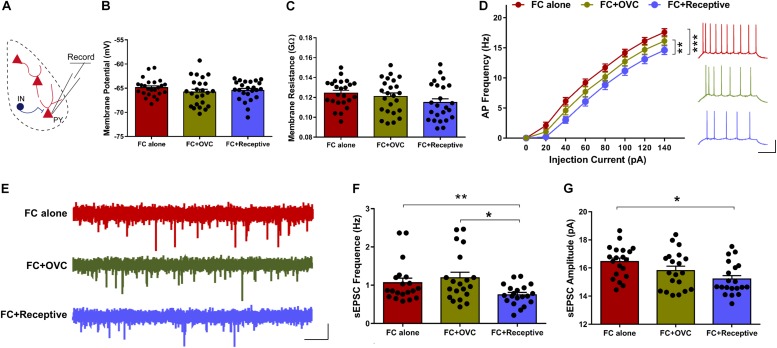
Decreased neuronal excitability and basal level of excitatory synaptic transmission in mice co-housed with a receptive mating partner. **(A)** Recording diagrams. Pyramidal neurons in the BLA were recorded in a whole-cell configuration. **(B)** Unaltered resting membrane potential of pyramidal neurons in the BLA. *n* = 24 neurons from five mice for each group. Student’s *t*-test, *P* > 0.05. **(C)** Similar membrane input resistance of pyramidal neurons in the BLA. *n* = 24 neurons from five mice for each group. Student’s *t*-test, *P* > 0.05. **(D)** Firing rate plotted against gradually increased currents. *n* = 24 neurons from five mice for each group. Two-way ANOVA, group effect, **P < 0.01. Right: representative traces of spikes in BLA pyramidal neurons evoked by injecting depolarizing currents of 80 pA. Scale bars: 20 mV, 200 ms. **(E)** Representative sEPSC traces of excitatory neurons in the BLA. Scale bar: 10 pA, 2 s. **(F)** Decreased sEPSC frequency of excitatory neurons in the BLA of mice in the “FC + Receptive” group. *n* = 20 neurons from five mice for each group. Student’s *t*-test: “FC + Receptive” vs. “FC alone”: *P = 0.048; “FC + Receptive” vs. “FC + OVC”: *P = 0.016. **(G)** Comparative sEPSC amplitude in BLA pyramidal neurons in all three groups. *n* = 20 neurons from five mice for each group. Student’s *t*-test, “FC + Receptive” vs. “FC alone”: *P* = 0.430; “FC + Receptive” vs. “FC + OVC”: *P* = 0.348.

It is known that approximately 85% of the neuronal population in the BLA are excitatory neurons (Mcdonald, 1992). In light of how the co-localization rate of cFos and PV was low and not found to be significantly different among the three groups (Figure 2D), we hypothesized that it is the excitatory neurons whose excitability is reduced when the male mice were co-housed with a receptive mating partner prior to fear extinction. To examine this hypothesis, we recorded the excitatory neurons in a whole-cell configuration using a patch-clamp technique (Figure 3A). While the resting membrane potential and membrane resistance were not changed among the three groups (Figures 3B,C; Student’s *t*-test, for resting membrane potential, “FC + Receptive” vs. “FC alone,” *t*(46) = 1.031, *P* = 0.308; “FC + Receptive” vs. “FC + OVC,” *t*(46) = 0.4653, *P* = 0.6439; “FC + OVC” vs. “FC alone,” *t*(46) = 1.317, *P* = 0.1944; for membrane resistance, “FC + Receptive” vs. “FC alone,” *t*(46) = 1.957, *P* = 0.0565; “FC + Receptive” vs. “FC + OVC,” *t*(46) = 1.099, *P* = 0.2776; “FC + OVC” vs. “FC alone,” *t*(46) = 0.7562, *P* = 0.4534), the firing frequencies in response to the injected depolarizing currents with gradual increments in the intensities were significantly decreased in the “FC + Receptive” group compared with those in the “FC alone” and “FC + OVC” groups (Figure 3D, two-way ANOVA, group effect, “FC + Receptive” vs. “FC alone,” *F*_(__1_,_368__)_ = 56.82, *P* < 0.001; “FC + Receptive” vs. “FC + OVC,” *F*_(__1_,_368__)_ = 10.66, *P* = 0.0012). Note that the firing frequencies in the “FC + OVC” group were also decreased compared with those in the “FC alone” group (Figure 3D, two-way ANOVA, group effect, *F*_(__1_,_368__)_ = 16.95, *P* < 0.001), which is probably due to a general social buffering effect (not copulatory behavior) on neuron activity in the amygdala. Nevertheless, these observations suggest attenuated excitability of excitatory neurons in the BLA of mice that were accompanied by a receptive mating partner.

To further investigate the mechanisms of decreased neuronal excitability in the BLA excitatory neurons in mice that were accompanied by a receptive mating partner, we recorded sEPSCs (Figure 3E). The frequency of sEPSCs was decreased in “FC + Receptive” group when compared with that in the “FC alone” and “FC + OVC” groups (Figure 3F; Student’s *t*-test, “FC + Receptive” vs. “FC alone,” *t*(38) = 2.4, *P* = 0.0214; “FC + Receptive” vs. “FC + OVC,” *t*(38) = 2.828, *P* = 0.0074). In addition, the amplitude was also reduced in the “FC + Receptive” group compared with that in the “FC alone” groups (Figure 3G; Student’s *t*-test, *t*(38) = 3.462, *P* = 0.0013), although there was no significant difference between the “FC + Receptive” group and the “FC + OVC” groups (Figure 3G; Student’s *t*-test, *t*(38) = 1.543, *P* = 0.1311). Together, these results suggest an attenuated excitatory synaptic input in the amygdala, which may serve in part as the cause of decreased neuronal excitability and subsequent reduced neuronal activation in mice that is accompanied by a receptive mating partner.

## Discussion

In the present study, we examined the effect of social company by a receptive or non-receptive mating partner on fear extinction. We found that company by non-receptive mating partner exhibited little effect on the fear response, while company by a receptive one significantly reduced the fear response during both fear extinction trining and a retrieval test. The number of cFos-positive cells, but not PV-positive interneurons, was found to be decreased in the BLA of male mice that accompanied by a receptive mating partner. Electrophysiological studies suggested that the excitability of excitatory neurons in the BLA of male mice that accompanied by a receptive mating partner was reduced, which may be due to the attenuated basal level of excitatory synaptic transmission. Altogether, these observations revealed that social company by a receptive mating partner had a beneficial effect on the facilitation of fear extinction.

Exposure therapy combined with pharmacotherapy represents an effective treatment for PTSD patients. Nevertheless, there were still a number of patients (39%) who failed to respond positively to this therapy (Gutner et al., 2016). In addition, PTSD symptoms may relapse once the exposure therapy ends, resulting in fear reinstatement (Singewald et al., 2015). Thus, the development of a more effective treatment method is needed. It is noteworthy that a positive social environment, including the attachment to intimate partners, is usually associated with better prognosis during treatment (DiGangi et al., 2013; Ferrer-Perez et al., 2019). Numerous clinical and animal studies have demonstrated the beneficial effect of social buffering on alleviating symptoms of PTSD (Kiyokawa et al., 2007; Hori et al., 2018; Charlson et al., 2019). Specifically, a variety of animal studies had focused on the contributions of social support from a same-sex unfamiliar partner during fear extinction training or retrieval test. Beneficial effects of the sexual activity have also been reported by numerous studies on an individual’s emotional state, which include the reduction in the anxiety levels (Fernández-Guasti et al., 1989; Waldherr and Neumann, 2007) and the depressive-like behaviors (Martinez-Mota et al., 2005). In addition, attenuated neuronal stress responsiveness within the hypothalamic paraventricular nucleus and corticotrophin releasing hormone synthesis after mating behaviors were also reported (Waldherr et al., 2010). Bai et al. (2009) observed a reduced fear response in male rats when exposed to females for 24 h immediately after contextual fear conditioning, which was mediated by activation of dopaminergic D1/D5 receptor in the hippocampus. However, this study did not include an extinction phase, which mimics the exposure therapy for PTSD treatment and was thus unable to reveal the effect of sexual activity on fear extinction. The findings from the current study add to the gaps in knowledge from the previous studies by showing that social company by a receptive female, but not an ovariectomized female, can promote tone-cued fear extinction in male mice, which is likely caused at least partially by decreased activation of neurons and excitatory synaptic transmission in the amygdala. A seemingly inconsistent result with the previous report is that similar freezing levels in response to the first tone (S1) during extinction training among the three groups were found in our study, indicating the limited effect of sexual activity on fear memory formation/consolidation. While the exact reason for the discrepancy is not clear, we speculate that it may be attributed to the different species (rat vs. mice), the types of fear memory (contextual vs. tone-cued), and the differences in the intensity of fear conditioning (three pairs of CS–US, 1 mA, and 1 s for US vs. six pairs of CS–US, 0.7 mA, and 2 s for US). It is noteworthy that the intensity of fear conditioning training in our study is so strong that it not only induced robust freezing response, but the fear level was also hard to decrease following fear extinction training (Mikami et al., 2016). Additional thoughts were also given to the question of whether the beneficial effect of co-housing with a receptive mating partner is due to sexual copulating behavior or just social contact (non-sexual behavior). Although the social interaction and sexual behaviors, including odor sniffing, ultrasonic communication, pursuit, mounting, and mating, were not quantified in detail during co-housing, we confirmed their sexual behaviors through the presence of the vaginal plugs in the receptive female mice. Not surprisingly, we found that the majority of receptive female mice in the “FC + Receptive” group had the vaginal plugs (Byers et al., 2012). We found that none of the non-receptive female mice in the “FC + OVC” group had the vaginal plug. While the absence of a vaginal plug could not necessarily exclude the mating behavior, our pooled data derived from all the mice in “FC + Receptive” group revealed a positive correlation between sexual behaviors and the acquisition of fear extinction. It is worth noting that additional alternative approaches with quantifiable parameters are required in the future study to examine the mating behavior and its effect on fear memory to further verify and strengthen our observations. Nevertheless, to our best knowledge to date, our study is the first study to investigate the influence of social company by a mating partner on fear extinction.

Numerous studies indicate that the BLA region of the amygdala is critical for acquisition of extinction memory (LaBar et al., 1998; Lu et al., 2001; Walker et al., 2002). It has been reported that the cFos expression, a marker of neuronal activity, was found to be increased in the basal regions of the amygdala in rodent animals following fear extinction when compared with those without fear conditioning or without extinction (Herry and Mons, 2004; Knapska and Maren, 2009). However, there are studies that had reported contradicting results, showing that neuronal activity is silenced in basal amygdala following fear extinction (Trouche et al., 2013; Maeng et al., 2017). Although the exact reasons underlying the discrepancy are still not clear, we presumed that it was at least in part due to different timing of cfos staining or distinct extinction procedures. In our study, we found a reduced number of cFos-positive neurons in the BLA of mice co-housed with a receptive mating partner, which may indirectly suggest that sexual activity may reduce neuron activation in response to fear extinction training. In addition, sparse colocalization of cFos and PV was also observed in BLA, which suggsets that the decreased density of cFos-positive neurons was not due to the alteration of PV-positive interneurons but may rather be due to that in the excitatory neurons. Indeed, our electrophysiological findings indicated reduced excitability of excitatory neurons in the BLA region, which may be caused by the decreased basal level of excitatory neurotransmission. Nevertheless, we cannot fully exclude the possibility of the involvement from another types of interneurons.

It is not clear how sexual behavior modulates neuronal function in the BLA and thus fear extinction. It is well known that sexual behavior, a deeply rooted physiological condition conserved throughout the species, is regulated by the nervous, endocrine, and genital systems whose interplay ensures its proper occurrence (Calabro et al., 2019). In the central nervous system, both cortical and subcortical structures, including the hypothalamus, brainstem, and spinal cord, are involved in finely adjusting this primitive and complex behavior. Besides, multiple neurotransmitter systems, including dopaminergic, serotonergic, cholinergic, as well as other neuropeptide transmitter systems, appear to have tremendous influence on the diverse aspects of this sexual response (Maejima et al., 2013; Burkett et al., 2016; Locklear et al., 2016). It has been reported that pleasurable behaviors, including sexual activity, reduce stress and induce structural plasticity in the BLA (Ulrich-Lai et al., 2010). Moreover, changes in brain activity in the amygdala have been observed in the human patients with non-organic erectile dysfunction (Chen et al., 2017). The expression level of tyrosine hydroxylase and the dopamine D2 receptor in BLA was also decreased in the animal model in which the infusion of a dopamine D2 receptor agonist can reverse the sexual phenotypes (Chen et al., 2019). Hence, these results suggest the importance of the dopaminergic signaling in the BLA for the regulation of sexual activity. On the other hand, it has also been reported that dopaminergic activity is elevated by sexual activity in mice and rats (Becker et al., 2001; Kudwa et al., 2005). A growing literature indicates that dopamine plays an important role in fear extinction (Abraham et al., 2014). A previous study reported that activation of D2 receptors facilitates fear extinction and that blockade of D2 receptors impairs fear extinction (Shi et al., 2017). Furthermore, a blockade of the D1 receptor in the BLA before an extinction session disrupted fear extinction (Hikind and Maroun, 2008). In light of the result that a reduced fear response was found in male rats when exposed to females for 24 h immediately after contextual fear conditioning, which was mediated by activation of dopaminergic D1/D5 receptor in the hippocampus (Bai et al., 2009), a plausible prediction is that sexual activity facilitates fear extinction probably through alteration of dopaminergic activity in the BLA. Nevertheless, further research is necessary to verify whether dopaminergic signaling is involved in the regulation of fear extinction by sexual behavior.

## Data Availability Statement

The datasets generated for this study are available on request to the corresponding author.

## Ethics Statement

The animal study was reviewed and approved by the Animal Ethics Committee of the Guangzhou Medical University.

## Author Contributions

FG wrote the first draft of the manuscript. X-DS edited the manuscript. X-DS, AX, and FG designed the research. FG, JH, Y-FG, G-BH, W-JL, X-YH, and Z-CQ performed the research. Y-LZ, S-TZ, and JL analyzed the data.

## Conflict of Interest

The authors declare that the research was conducted in the absence of any commercial or financial relationships that could be construed as a potential conflict of interest.
